# Discovery and characterization of a thermostable two-domain GH6 endoglucanase from a compost metagenome

**DOI:** 10.1371/journal.pone.0197862

**Published:** 2018-05-24

**Authors:** Marianne S. Jensen, Lasse Fredriksen, Alasdair K. MacKenzie, Phillip B. Pope, Ingar Leiros, Piotr Chylenski, Adele K. Williamson, Tony Christopeit, Heidi Østby, Gustav Vaaje-Kolstad, Vincent G. H. Eijsink

**Affiliations:** 1 Faculty of Chemistry, Biotechnology and Food Science, Norwegian University of Life Sciences, Ås, Norway; 2 The Norwegian Structural Biology Centre, Department of Chemistry, UiT-The Arctic University of Norway, Tromsø, Norway; Institut National de la Recherche Agronomique, FRANCE

## Abstract

Enzymatic depolymerization of recalcitrant polysaccharides plays a key role in accessing the renewable energy stored within lignocellulosic biomass, and natural biodiversities may be explored to discover microbial enzymes that have evolved to conquer this task in various environments. Here, a metagenome from a thermophilic microbial community was mined to yield a novel, thermostable cellulase, named mgCel6A, with activity on an industrial cellulosic substrate (sulfite-pulped Norway spruce) and a glucomannanase side activity. The enzyme consists of a glycoside hydrolase family 6 catalytic domain (GH6) and a family 2 carbohydrate binding module (CBM2) that are connected by a linker rich in prolines and threonines. MgCel6A exhibited maximum activity at 85°C and pH 5.0 on carboxymethyl cellulose (CMC), but in prolonged incubations with the industrial substrate, the highest yields were obtained at 60°C, pH 6.0. Differential scanning calorimetry (DSC) indicated a T_m(app)_ of 76°C. Both functional data and the crystal structure, solved at 1.88 Å resolution, indicate that mgCel6A is an endoglucanase. Comparative studies with a truncated variant of the enzyme showed that the CBM increases substrate binding, while not affecting thermal stability. Importantly, at higher substrate concentrations the full-length enzyme was outperformed by the catalytic domain alone, underpinning previous suggestions that CBMs may be less useful in high-consistency bioprocessing.

## Introduction

Cellulose is a major component of plant cell walls, where it contributes to the rigidity that enables the plant to stretch towards the sunlight. Estimates of worldwide annual plant biomass production amount to a staggering 170–200 billion tons, of which approximately 70% is cell wall material often referred to as lignocellulose [1]. Accordingly, lignocellulose represents the most abundant biomass on Earth and thus our largest renewable carbon reservoir, and holds great potential for sustainable energy production [2, 3]. Utilization of lignocellulose as a “green carbon” feedstock to replace oil-based commodities can generate second-generation biofuels and higher-value products from low-value waste products in agriculture and forestry, and efficient enzymatic depolymerization is a key step in such biomass-based value creation [4–6].

Lignocellulosic biomass is composed of a complex matrix of polysaccharides (cellulose and various hemicellulose) and the aromatic polymer lignin. The ratio of these compounds differs between plant species, but generally amounts to 40–50% cellulose, 20–35% hemicellulose and 15–30% lignin [1]. Cellobiose is the repeating unit of cellulose, and consists of two glucose units rotated 180° relative to one another and joined by a β-1,4 glycosidic linkage [7]. Individual cellulose chains arrange into crystalline fibrils that are stabilized by extensive hydrogen bonding, and in the plant cell wall these fibres are embedded in a matrix of hydrophobic lignin and amorphous hemicelluloses [8, 9]. As a result, lignocellulose constitutes a recalcitrant structure that is highly resistant to degradation.

Microorganisms have evolved the ability to degrade lignocellulose by producing specialized enzymes. Various catalytic activities with different roles in lignocellulose conversion have been identified, reflecting the complexity and diversity of naturally occurring substrates. Deconstruction of the most recalcitrant of the polysaccharides, cellulose, is achieved by cellulases, which are hydrolytic enzymes belonging to various glycoside hydrolase (GH) families in the Carbohydrate Active enZymes database (CAZy; www.cazy.org). Cellulases are usually classified further as endoglucanases, which cleave internal glycosidic bonds, and cellobiohydrolases, which release cellobiose from accessible reducing or non-reducing chain ends. The cellobiose is finally converted to glucose by β-glucosidases [10]. Efficient degradation of lignocellulose requires additional enzymes including lytic polysaccharide monooxygenases (LPMOs), which employ an oxidative mechanism to break internal bonds in crystalline cellulose regions [11–15], and hemicellulases that degrade various hemicelluloses (e.g. xyloglucans, xylans, mannans and glucomannans) surrounding the cellulose fibrils [9]. All the enzymes involved may contain one or more carbohydrate binding modules (CBMs), which are thought to increase the effective enzyme concentration on the substrate surface by positioning the catalytic domains in proximity to the substrate [16, 17].

Due to the recalcitrance of lignocellulosic biomass, thermochemical pre-treatments (e.g. steam explosion or acidic/alkaline treatments) are implemented prior to enzymatic treatments, to accelerate industrial lignocellulose turnover in biorefineries. Such pre-treatments often require harsh conditions, which may lead to destruction of sugars and the production of various enzyme inhibitory substances [6, 18]. Because of this, and because it is generally considered favourable to carry out the enzymatic conversion processes at higher temperatures [19], there is a need for robust enzymes that originate from harsh natural environments, or that have been made robust by enzyme engineering. Today’s commercial market for cellulases is dominated by fungal enzymes from species such as the mesophilic fungus *Trichoderma reesei* [4, 20]. The temperature optima of many fungal cellulases are in the 40–50°C range, which is lower than what is commonly desired in industry [21]. Even today, enzyme costs remain a significant factor in industrial biomass processing [22, 23], so there is a general interest in discovering better enzymes.

In an attempt to find thermostable enzymes for cellulose processing, we have explored the metagenome of a thermostable microbial community derived from rice straw inoculated with compost and incubated at 55°C [24]. This led us to express and characterize a 45 kDa two-domain thermostable bacterial cellulase comprised of a GH6 domain and a C-terminal CBM2 domain. We present the functional and structural characteristics of this enzyme, called mgCel6A, and assess its potential for use in high-temperature industrial degradation of sulfite-pulped lignocellulosic biomass (Norway spruce). With a view on the potential industrial application of mgCel6A, we have also assessed the role of the CBM and how this role depends on the dry matter concentration in the enzymatic reaction.

## Materials and methods

### Enzyme origin and homology modelling

Publicly available metagenome data accessible in the Joint Genome Institute IMG/M database (https://img.jgi.doe.gov/cgi-bin/m/main.cgi; IMG genome ID 2199352008) were mined for putatively lignocellulose active enzymes using dbCAN (csbl.bmb.uga.edu/dbCAN; [25]). The 1404 bp mgCel6A encoding gene (IMG gene ID 2200387098) was extracted from the metagenome, and was annotated using InterPro (www.ebi.ac.uk/interpro). BLASTp (blast.ncbi.nlm.nih.gov) and the PDB database (rcsb.org) were used to investigate similarities to known cellulases and to check for occurrence of expected active site residues, and structures of homologous proteins were visualized and inspected in PyMol (pymol.org).

### Cloning, expression and purification of mgCel6A and mgCel6AΔCBM

The *mgCel6A* gene (codon-optimized for *Escherichia coli* expression) was synthesized (Thermo Fisher Scientific, Waltham, Massachusetts, USA) and bp 106–1404 (omitting the predicted 35 amino acid signal peptide sequence) was amplified by PCR using Phusion DNA polymerase (New England Biolabs, Ipswich, Massachusetts, USA) and suitable primers (Eurofins, Ebersberg, Germany). To facilitate further subcloning, the forward primer was 5'TAAGAAGGAGATATACTATGGCAGATAGCGCATTTTATGTTGAT3' where the underlined nucleotides represent an over-hang sequence for ligation-independent cloning (LIC; [26]). Two different reverse primers were employed, one to amplify the full-length gene (*mgcel6A*) with sequence 5'AATGGTGGTGATGATGGTGCGCGCTGGTACATGCACTACCATTCAG3', and one to amplify the catalytic domain alone (*mgcel6AΔCBM*) with sequence 5'AATGGTGGTGATGATGGTGCGCTGCTGCAATTGCCAGTTCATAT3'. The PCR products were purified using a Nucleospin Gel and PCR Clean-Up kit (Macherey-Nagel, Düren, Germany) and inserted into the pNIC-CH expression vector (AddGene, Cambridge, Massachusetts, USA) by LIC. As a result of this cloning strategy, the N-terminus of the (signal peptide-free) protein is extended with a methionine, while a seven residue His-tag (AHHHHHH) is added at the C-terminus. LIC was followed by heat shock transformation into chemically competent OneShot *E*. *coli* TOP10 cells (Invitrogen, Carlsbad, California, USA). The host strain was allowed to proliferate in Super Optimal broth with Catabolite repression (SOC) for 60 minutes prior to plating on Lysogenic Broth (LB) agar containing 50 μg/ml kanamycin and 5% sucrose, followed by incubation overnight at 37°C. Single transformant colonies were inoculated in liquid LB containing 50 μg/ml kanamycin and incubated overnight at 37°C. The plasmid was isolated from transformants using a NucleoSpin Plasmid kit (Macherey-Nagel), and the cellulase-encoding gene sequences were verified by Sanger sequencing (GATC, Konstanz, Germany). The isolated plasmids were subsequently transformed by heat shock into chemically competent OneShot BL-21 Star^TM^ (DE3) *E*. *coli* cells (Invitrogen) and grown in SOC media as described above, before plating on LB agar containing 50 μg/ml kanamycin and overnight incubation at 37°C. Transformant colonies were inoculated and grown in Terrific Broth (TB) containing 50 μg/ml kanamycin using a Harbinger system (Harbinger Biotechnology & Engineering, Markham, Canada) at 22°C overnight. Protein expression was subsequently induced by addition of 0.2 mM isopropyl-β-D-thiogalactopyranoside (IPTG) and the cultures were incubated for another 24 hrs at 22°C. The cell pellets were harvested by centrifugation at 5000 x *g* for 15 minutes (Beckman Coulter Brea, California, USA), followed by rapid cooling to minus 80°C, after which the cells were resuspended in 50 mM Tris pH 8.0 with 500 mM NaCl and 5 mM imidazole. The cells were lysed using a Vibracell sonicator (Sonics & Materials Inc., Newtown, Connecticut, USA) with 5 seconds on/off pulses for 3 minutes at 30% amplitude while kept on ice, and the cell debris was removed by centrifugation at 15,000 x *g* for 15 minutes. The cell-free protein extracts were filtrated using 0.45 μm syringe filters (Sarstedt, Nümbrecht, Germany) after which the proteins were purified by immobilized metal affinity chromatography (IMAC) using an Äkta pure chromatography system (GE HealthCare, Chicago, USA) equipped with a Ni^2+^ affinity HisTrap^TM^ HP 5 ml column (GE HealthCare). The His-tagged proteins were eluted using a linear gradient of 5–500 mM imidazole in 50 mM Tris pH 8.0, 500 mM NaCl. Protein fractions were examined by SDS-PAGE (Bio-Rad, Hercules, California, USA), after which relevant fractions were pooled and concentrated using 10,000 MWCO (Molecular Weight Cut-Off) Vivaspin ultrafiltration tubes (Sartorius, Göttingen, Germany), with simultaneous buffer exchange to 20 mM Tris-HCl, pH 8.0. The purified proteins were stored at 4°C. The protein concentration was estimated using the Bio-Rad Protein Assay (Bio-Rad) based on the Bradford method [27] or by measuring the A_280_ and using theoretical extinction coefficients (web.expasy.org/protparam) for calculating the concentrations. In both cases, a Biophotometer (Eppendorf, Hamburg, Germany) was used for measuring absorbance.

### Crystallization, data collection, structure determination, and model refinement

Crystallization experiments were performed with a stock solution of the (His-tagged) GH6 catalytic domain (12 mg/ml, estimated by A_280_) in 20 mM Tris-HCl pH 8.0. Initial crystallization conditions were screened using the vapour diffusion sitting drop method using a Phoenix crystallization robot (Art Robbins Instruments, Sunnyvale, California, USA). The plates were set up with 60 μl reservoir solutions and sitting drops with equal amounts of reservoir solution and protein stock solution in a total drop volume of 1 μl. During incubation at 20°C, crystals appeared after about 5 weeks at conditions containing 1 M (NH4)_2_SO_4_, 0.1 M BisTris pH 5.5, and 1% PEG 3350. Crystals were harvested, transferred to a cryoprotectant solution consisting of the reservoir solution containing 27% Ethylene glycol, and subsequently flash cooled in liquid N_2_. X-ray diffraction data were collected at the European Synchrotron Radiation Facility (ESRF; Grenoble, France) beamline ID30B. Data collection and processing statistics are presented in Table 1. The crystal structure was solved by molecular replacement using MolRep in the CCP4 program package [28] with 2boe as a search model (this is a single mutant of Cel6A from *Thermobifida fusca*; this is the *Tf*Cel6A structure with the highest available resolution, 1.15 Å; [29]). The initial refinement was executed in Refmac [30] followed by automated model improvement in Buccaneer [31]. The manual building was done in Coot [32] interspersed by cycles of refinement in Refmac and resulted in a final R_work_/R_free_ of 15.83/20.44. The atomic coordinates and structure factors have been deposited in the RCSB Protein Data Bank (PDB; www.rcsb.org) with accession code 6FAO (S1). Figs presented in the results section were generated using Pymol (pymol.org). The DALI server (http://ekhidna2.biocenter.helsinki.fi/dali) was used to generate a structure-based alignment of mgCel6A (PDB ID: 6FAO), the homologous endoglucanase *Tf*Cel6A (PDB ID: 1TML; [33]), and the cellobiohydrolase *Tf*Cel6B (PDB ID: 4B4H; [34]). Residues missing from the PDB files due to poorly defined electron density were manually inserted in the structurally aligned sequences based on visual inspection of superimposed structures in Pymol. ESPript [35] was employed to visualize the final structure-based alignment and highlight features such as conserved residues and specific loop regions.

**Table 1:** Data collection and refinement statistics.

Space group	*P*2_1_2_1_2_1_
Wavelength (Å)	0.976
Resolution range (Å)	57.91–1.88 (1.92–1.88)
Completeness (%)	99.3 (94.4)
Mean I/σ(I)	10.5 (1.8)
R_p.i.m._	0.064 (0.534)
Total No. of reflections	135306 (6962)
No. of unique reflections	27338 (1636)
R_work_/R_free_	15.83/20.44
**Cell dimensions**	
a, b, c (Å)	48.74, 78.68, 85.53
α, β, γ (°)	90, 90, 90
**Number of non-H atoms**	
Protein	2108
Water	379
Other*	9
Total	2496
**R.m.s. deviations**	
Bonds (Å)	0.019
Angles (°)	1.738
**Average B factors (Å**^**2**^**)**	
Overall	17.34
Protein	16.60
Water	29.23
Other^a^	29.50
**Ramachandran plot (# / %)**	
Preferred	258 / 93.48
Allowed	17 / 6.16
Outliers^b^	1 / 0.36

Values in parentheses are for the outermost shell.

^a^A sulfate ion and one molecule of ethylene glycol were identified in electron density and added. The sulfate ion was likely introduced during crystallisation and the ethylene glycol molecule was probably introduced due to cryo protection.

^b^Residues in the Ramachandran plot were categorized according to the nomenclature used in WinCoot version 0.8.6.1.

### Apparent melting temperature (T_m(app)_)

A Nano-Differential Scanning Calorimeter III (Calorimetry Sciences Corporation, Lindon, USA) was employed to determine the apparent melting temperatures of mgCel6A and mgCel6AΔCBM. The sample solutions contained approximately 1.5 mg/ml enzyme dialyzed overnight at 4°C against 75 mM phosphate-citrate buffer, pH 6.0, and were degassed (5 min) and filtered (0.22 μm) prior to loading the sample cell. Buffer from the dialysis, also degassed and filtered, was used to record buffer baselines prior to the protein scans. A scan rate of 1°C/min from 20°C to 90°C was employed, and the experiments were carried out in duplicate, using freshly dialyzed enzyme for each scan. The data were analysed using the NanoAnalyze software (tainstruments.com); buffer baselines were subtracted from the protein scans.

### Substrates

Enzyme activity was primarily evaluated on an industrial substrate derived from unbleached Norway spruce (*Picea abies*) through a sulfite pulping pre-treatment termed the BALI^TM^ process [36, 37], developed at Borregaard AS (Sarpsborg, Norway). The substrate had a glucan content of 88%, while hemicelluloses and acid insoluble lignin comprised the remaining 12%. The substrate was dried at 40°C overnight and the particle size was reduced in a planetary ball mill PM 100 (Retsch, Haan, Germany) followed by sieving through a 0.85 mm screen, to make the substrate amenable to use in small-scale reactions. Enzyme activity was also assessed on the cellulosic model substrates carboxymethyl cellulose (CMC) (Sigma-Aldrich, St. Louis, Missouri, USA), Avicel PH-101 (Sigma-Aldrich), filter paper (Whatman no.1) and phosphoric acid swollen cellulose (PASC) prepared from Avicel PH-101 according to [38], as well as on the hemicellulosic substrates konjac glucomannan (KGM), xylan and tamarind xyloglucan (all from Megazyme, Wicklow, Ireland). Cello-oligomers with degree of polymerization (DP) DP2-DP6 (Megazyme) were used as substrates for evaluating cleavage patterns.

### Activity assays

Phosphate-citrate buffers in the pH range pH 3.0–8.0 were used in all activity assays. Activity assays were carried out in 96-well microtiter plates (Thermo Fisher Scientific) with plastic sealing for short incubations, and in 2 mL screw cap micro tubes (Sarstedt) for overnight incubations. The reaction mixtures were incubated in thermomixers (Eppendorf, Hamburg, Germany), and the enzyme concentration in activity assays was 1 μM unless stated otherwise. Reactions with insoluble substrates were stopped by boiling the samples for 10 minutes, and the soluble reaction products were collected by vacuum filtration using 0.45 μm 96-well filter plates (Merck Millipore, Darmstadt, Germany). Reactions with soluble substrates were stopped by addition of a double volume of 3,5-dinitrosalicylic acid (DNS reagent) or an equal volume of 0.1 M NaOH depending on the method used for product analysis (DNS or HPLC, respectively). Hydrolysis yields in reactions were analysed using the DNS reagent for detection of reducing ends [39], and standard solutions of cellobiose (the main end-product generated by mgCel6A from cellulose) were used for quantification. Experiments were performed in triplicates.

### Optimal conditions

The optimal conditions for hydrolysis were initially assessed with 1% (w/v) CMC as substrate. The optimal temperature for activity was determined by comparing the yields from 10 minute incubations at temperatures ranging from 50°C to 90°C, and the optimal pH for activity was determined by comparing the yields from 10 minute incubations at pH 3.0–8.0. The optimal conditions for hydrolysis of the industrial sulfite-pulped spruce substrate were estimated in the same manner, using two different incubation times (15 minutes and 24 hours).

### Thermal and pH stability

To assess thermostability and tolerance to acidic pH, the enzyme was pre-incubated at 4 μM concentration for up to 24 hours in phosphate-citrate buffers ranging from pH 4.0 to pH 6.0, at 60°C, 65°C or 70°C. The enzyme samples were kept at 4°C after pre-incubation. The residual activities at various time points were estimated by diluting the pre-incubated enzyme two-fold in 2% (w/v) CMC in water followed by 10 minutes of hydrolysis at the optimal temperature (85°C) for the enzyme. The pHs were not adjusted after pre-incubation. Half-lives were determined by estimating the incubation time needed to reduce activity by 50%, compared to the activity of non-pre-incubated enzyme at the same pH.

### Reactions with various substrates

Degradation of various substrates (CMC, Avicel, PASC, sulfite-pulped spruce, KGM, xylan, and tamarind xyloglucan), all at 1% (w/v) in phosphate-citrate buffer pH 6.0 by 1 μM mgCel6A was examined by incubating reaction mixtures at 60°C and 600 rpm for 72 hours.

To determine the distribution of reducing ends in the soluble versus insoluble fraction after hydrolysis of cellulose, a filter paper assay based on the method described by Irwin et al. [40] was used. Disks of filter paper (4.0 mg, generated by a paper punch) were incubated with enzyme (0.5 μM) for 24 hours at 60°C in phosphate citrate buffer, pH 6.0, before the soluble fraction (supernatant) and insoluble fraction (remaining filter paper) were separated by centrifugation (12.000 x *g* for 5 minutes). 400 μl of the supernatant was removed and boiled with DNS reagent. The remaining filter paper was washed five times in distilled water, after which it was resuspended in 400 μl of distilled water and boiled with DNS reagent.

The efficiency of 1 μM mgCel6A or mgCel6AΔCBM in degrading sulfite-pulped spruce at varying dry matter (DM) loading (0.5–10% DM) was assessed after 48 hrs of incubation at 60°C and 1000 rpm. The conversion of 10% DM sulfite-pulped spruce by mgCel6A was determined with an enzyme loading of 8 mg enzyme per g cellulose for 72 hours at 1000 rpm, and the product yield was calculated according to Kristensen et al. [41]. The reactions were stopped by boiling the samples for 10 minutes, and the soluble products were separated from the insoluble substrates by vacuum filtration, as above. Activity on soluble cello-oligomers (DP2-6) was assessed using substrate concentrations of 0.1% (w/v) and an enzyme concentration of 1 μM; reaction mixtures were incubated at 60°C for 18 hrs without shaking and the reactions were stopped by addition of an equal volume of 0.1 M NaOH. Products were stored at 4°C until analysis by DNS [39], HPAEC-PAD, or MALDI-TOF MS (see below).

### Product analysis by HPAEC-PAD

Glucose and soluble cello-oligomers (DP2-6) were analysed by high-performance anion-exchange chromatography (HPAEC) using an ICS3000 system (Dionex, ThermoScientific, San Jose CA, USA) equipped with pulsed amperometric detection (PAD) and a CarboPac PA1 column (Dionex). A multistep linear gradient was used to elute the products at 0.25 ml/min, going from 0.1 M NaOH to 0.1 M NaOH, 0.1 M sodium acetate (NaOAc) in 10 minutes, to 0.1 M NaOH, 0.14 M NaOAc in 5 minutes, to 0.1 M NaOH, 0.3 M NaOAc in 1 minute, and to 0.1 M NaOH, 1.0 M NaOAc in 2 minutes, before column reconditioning by applying 0.1 M NaOH for 11 minutes. Data collection and analysis were carried out with the Chromeleon 7.0 software, and a DP1-DP6 cello-oligomer standard was used to quantify the products.

### Product analysis by MALDI-TOF MS

Product formation from hemicellulosic substrates was assayed qualitatively using an matrix-assisted laser desorption/ionization time-of-flight (MALDI-TOF) UltrafleXtreme mass spectrometer (Bruker Daltonics GmbH, Bremen, Germany) equipped with a Nitrogen 337-nm laser. Reaction products (1 μl) were applied to an MTP 384 ground steel target plate TF (Bruker Daltonics) together with 2 μl of 9 mg/ml of 2,5-dihydroxybenzoic acid (DHB) dissolved in 30% acetonitrile, followed by air-drying. Spectra were collected using Bruker FlexControl software and analysed with Bruker flexAnalysis software.

### Substrate binding

Enzyme binding to Avicel was examined using a slightly modified variant of the A_280_ method described by Vaaje-Kolstad and co-workers [42]. The binding mixtures contained 1% (w/v) Avicel and 0.1 mg/ml mgCel6A or mgCel6AΔCBM in phosphate-citrate buffer pH 6.0 and were incubated at 22°C and 1000 rpm. Substrate binding was monitored by determining the A_280_ of the liquid fraction at various time points, using aliquots that were taken from the binding reactions and vacuum filtered over a 0.45 μm filter to separate unbound protein from protein bound to substrate. Protein concentrations were calculated by using theoretical extinction coefficients.

## Results and discussion

### Sequence analysis and enzyme production

**Fig 1:**
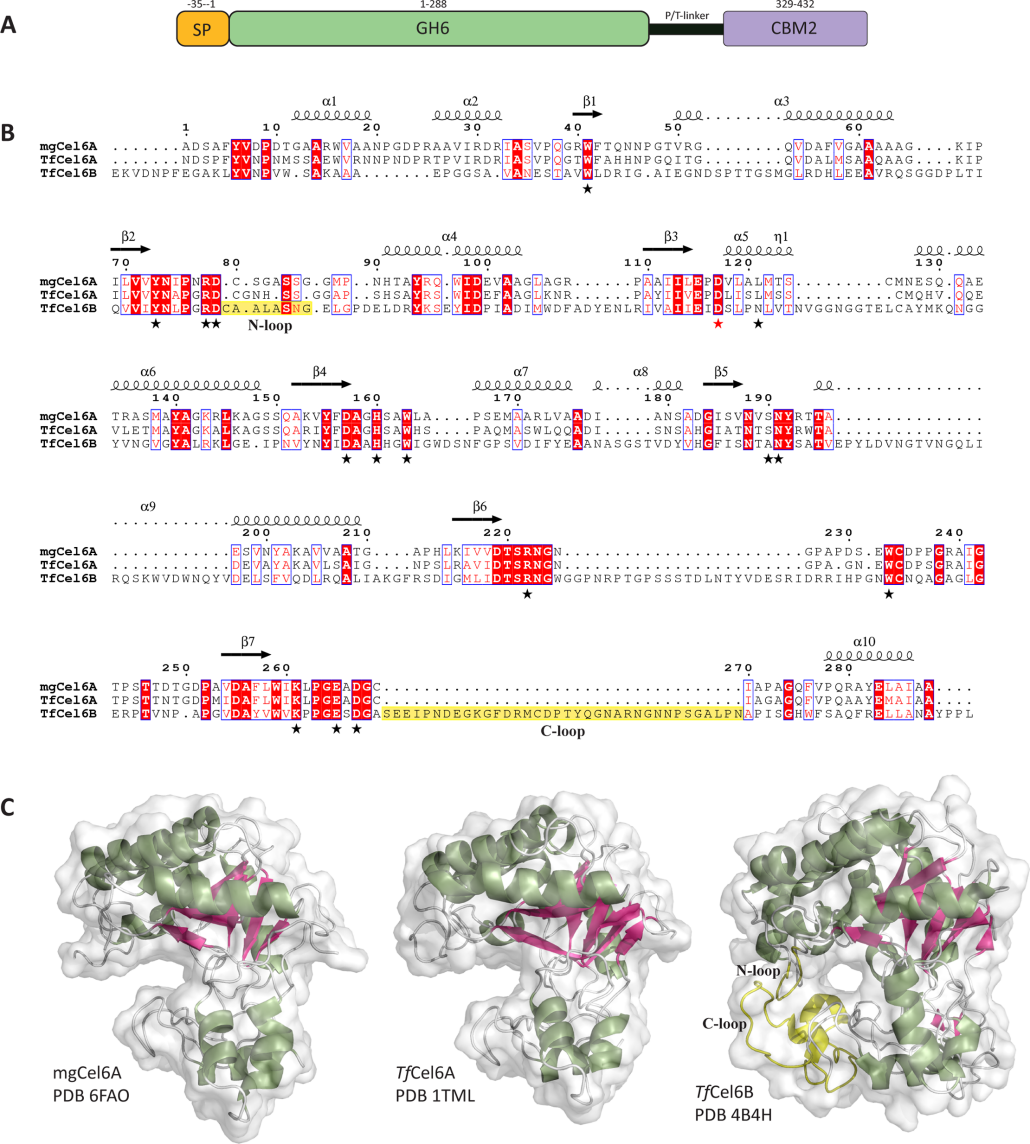
Sequence and structure of mgCel6A. (A) The domain structure of mgCel6A including a 35 residue signal peptide (SP), a 288 residue catalytic domain (GH6), a 40 residue proline- and threonine-rich linker, and a 104 residue carbohydrate binding module family 2 (CBM2). (B) Structure-based sequence alignment of the catalytic domains of mgCel6A (PDB ID: 6FAO), *Tf*Cel6A (PDB ID: 1TML), and *Tf*Cel6B (PDB ID: 4H4B). The secondary structure elements of mgCel6A are depicted above the aligned sequences. Red boxes show residues that are conserved in the three enzymes, while blue boxes indicate similar residues. A red star marks the catalytic acid, while black stars denote other residues potentially involved in catalysis and/or substrate-binding [29, 46, 47]; all these are shown in Fig 2A, 2C and 2E. Residues highlighted in yellow indicate two loops that differ between endocellulases and cellobiohydrolases in the bacterial GH6 family (see text for more details). (C) Side-view of the catalytic domain of mgCel6A (PDB ID: 6FAO), *Tf*Cel6A (PDB ID: 1TML), and *Tf*Cel6B (PDB ID: 4B4H), coloured by secondary structure (green α-helices and pink β-sheets), and with transparent surface representation. The amino-proximal (N) and carboxyl-proximal (C) loop regions of *Tf*Cel6B that are responsible for tunnel formation are displayed in yellow. Note that one end of the active site tunnel in *Tf*Cel6B can be closed off by a flexible loop that is not visible in the structure displayed here; for more details see [34].

Metagenome mining led to the identification of mgCel6A in metagenomic data originating from a high-temperature (55°C) rice straw/compost bioreactor. Studies have been published on the metagenome [24] and its metatranscriptome [43]. Studying this and a related mesophilic metagenome, Reddy and colleagues [24] showed that cellulases containing CBM2s, such as mgCel6A, were significantly overrepresented in the thermophilic rice straw/compost community. They also found that these CBM2-containing cellulase-encoding genes primarily belonged to the genus *Micromonospora*. Using the InterPro server, mgCel6A was predicted to comprise a signal peptide, a GH6 catalytic domain, a 40 residue proline- and threonine-rich linker, and a CBM2 (Fig 1A). Previously identified GH6 cellulases include both endoglucanases and cellobiohydrolases of mainly bacterial and fungal origin (CAZy). CBM2s are known to bind cellulose, and in some cases chitin or xylan [16].

Employing BLASTp, the closest relative of the GH6 domain was found to be an unpublished putative endoglucanase (KXK58956.1) from *Micromonospora rosaria*. The closest relative with a known structure in the PDB database is an endo-1,4-glucanase from *Thermobifidia fusca* (*Tf*Cel6A, formerly known as *Thermomonospora fusca* endocellulase E2; PDB ID: 1TML; 68% sequence identity; [33]). The PDB contains several more recent structures of *Tf*Cel6A, including a complex with a substrate analogue and single-mutation variants, with higher resolution (PDB ID: 2BOD, 2BOE, 2BOF, 2BOG; [29]).

The C-terminal CBM2 domain, which is connected to the GH6 domain by a 40-residue P/T-rich linker region (Fig 1A), has 67% sequence identity with the CBM of an unpublished endoglucanase from *Micromonospora echinaurantiaca* (SCG62086.1). As for homologues with known structure, the closest relative is a CBM associated with the bifunctional beta-1,4-xylanase/glucanase C_ex_ from *Cellulomonas fimi* (45% sequence identity; PDB ID: 1EXH; [44]). Notably, the 40-residue P/T linker in mgCel6A is long compared to its closest homologue with known structure, *Tf*Cel6A, where the catalytic domain and the CBM2 are connected by a 21-residue P/T/N linker.

Both mgCel6A and mgCel6AΔCBM were expressed in *E*. *coli*. SDS-PAGE showed that most of the protein was soluble after cell lysis, with only small remains in the cell pellet. The two proteins were easy to produce and purify and the yields of purified protein were approximately 500 mg and 350 mg per litre culture, respectively.

### Structure of the GH6 catalytic domain

The crystal structure of the catalytic domain of mgCel6A was solved to 1.88 Å (PDB ID: 6FAO) and is a typical representative of the seven-stranded TIM barrel α/β fold (Fig 1C). Statistics are shown in Table 1. The final model contained residues 2–288 of the expressed 288-residue catalytic domain plus an extra alanine from the C-terminal His-tag. Ser84 and Ser85 in a glycine-rich surface loop (SGASSGGM) could not be modelled.

**Fig 2:**
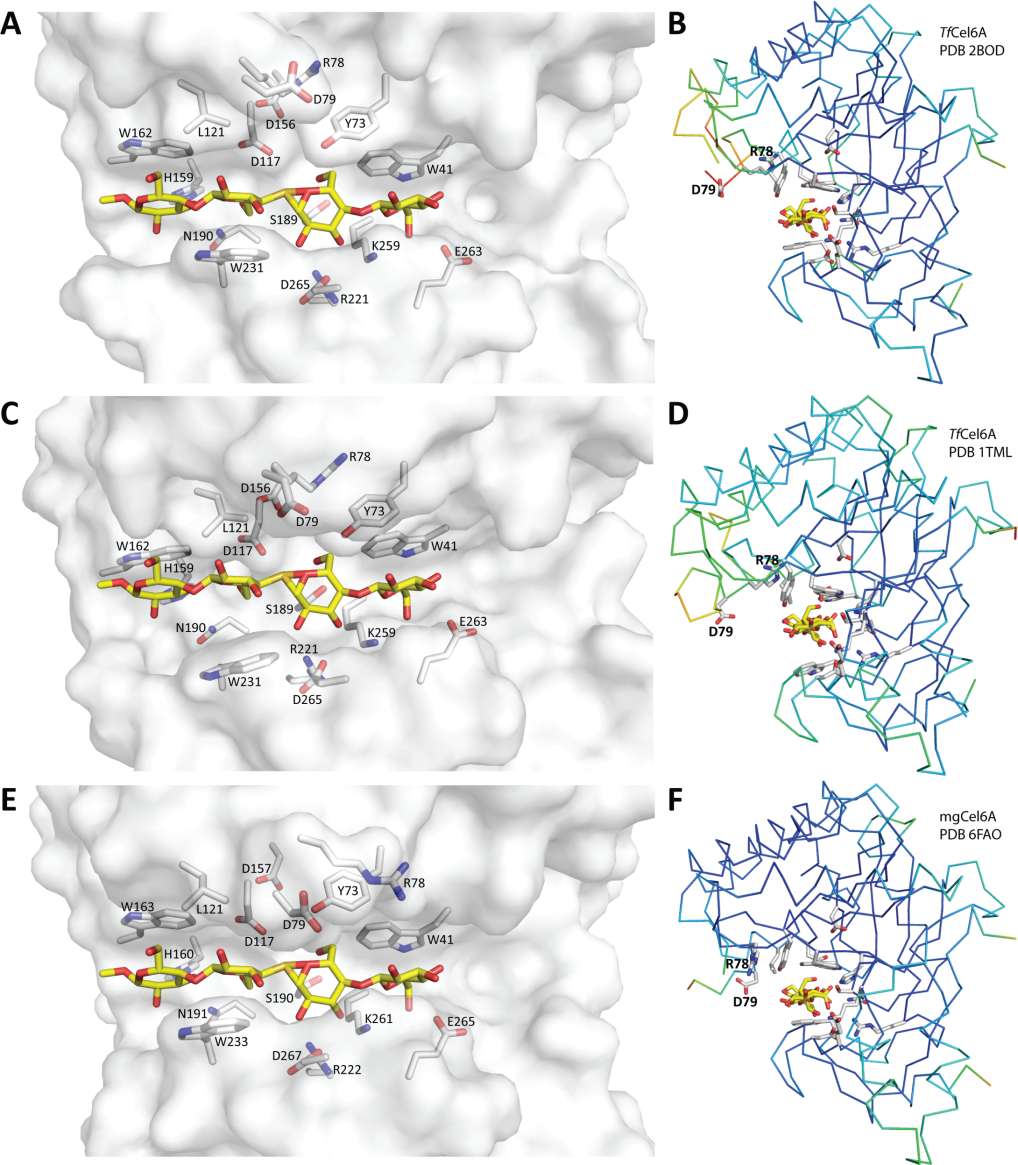
Comparison of the catalytic clefts of mgCel6A and *Tf*Cel6A. All pictures contain the substrate analogue methyl cellobiosyl-4-thio-beta-cellobioside which was modelled on the basis of superimposition with the crystal structure of *Tf*Cel6A in complex with this analogue (panel A, D; PDB ID: 2BOD), using Pymol. Panels A, C and E show transparent surface representations of the catalytic clefts of *Tf*Cel6A crystallized with ligand, apo *Tf*Cel6A with modelled ligand and apo mgCel6A with modelled ligand. The side chains of residues in the catalytic cleft (marked in Fig 1B) are represented by grey sticks, while the substrate analogue is represented by yellow sticks. Panels B, D and F show ribbon representations of the same three proteins, coloured according to B-factor (blue means low; red means high). Note that the N-loop lacks residues in two of the three structures, explaining the additional chain ends in this region (see text for details).

Fig 1B shows a structure-based sequence alignment of mgCel6A, the endocellulase *Tf*Cel6A (PDB ID: 1TML; [33]) and the cellobiohydrolase *Tf*Cel6B (PDB ID: 4B4H; [34]), highlighting conserved residues and the location of secondary structure elements in mgCel6A. Previous studies have shown that GH6 endoglucanases owe their open cleft structure to shortening and displacement of two surface loops compared to the corresponding loops in GH6 cellobiohydrolases. In the latter, elongated loops fold over the active site cleft and close it off to form a tunnel, which likely restricts the enzyme to attacking the chain ends of cellulose, while promoting processivity [45]. Fig 1B and 1C highlight two loop regions termed the N-loop (amino-proximal) and C-loop (carboxyl-proximal). The N-loop is highly flexible (Fig 2) in both mgCel6A and *Tf*Cel6A to the extent that two of its residues could not be modelled in the mgCel6A structure. The N-loop has a different conformation and is slightly extended in *Tf*Cel6B, which together with a drastically extended C-loop (Fig 1B) gives the active site of this enzyme a tunnel-like character (Fig 1C). MgCel6A has a deep but open substrate-binding cleft similar to the one observed in *Tf*Cel6A, which suggests that mgCel6A, like *Tf*Cel6A, is an endoglucanase.

Residues known to be involved in catalysis and exposed residues that shape the substrate-binding catalytic cleft of *Tf*Cel6A [29, 46, 47] are marked with stars in the structure-based sequence alignment of Fig 1B, which shows that most of these residues are conserved in mgCel6A and to a somewhat lesser extend in *Tf*Cel6B. Based on the analogy with *Tf*Cel6A, Asp117 is the catalytic acid in mgCel6A, while the catalytic base remains elusive, as is the case for GH6 cellulases in general [48].

Fig 2 presents a closer view on the catalytic clefts of structures of *Tf*Cel6A and mgCel6A and includes the structure of *Tf*Cel6A bound to the substrate analogue methyl cellobiosyl-4-thio-beta-cellobioside [29]. Generally, the clefts look similar except for Arg78 and Asp79 whose conformations vary between the three structures. Notably, these two residues are adjacent to the flexible N-loop (high B-factors; Fig 2B, 2D and 2F). Asp79 has been proposed as the catalytic base in *Tf*Cel6A [47], even though this aspartate does not reside within hydrogen-bonding distance of a water molecule that could act as a nucleophile on the scissile bond. Based on simulations of the cellobiohydrolase Cel6A from *Trichoderma reesei* (*Tr*Cel6A), Mayes and co-workers recently proposed that Asp175 in *Tr*Cel6A (corresponding to Asp79 in mgCel6A) may act as a more “remote” catalytic base by coordinating a short water wire that comprises two water molecules aligned between the scissile bond and the aspartate [49].

The variation in the conformations of Arg78 and Asp79 and the high B-factors of the N-loop clearly suggest that these residues and the N-loop change their conformation during substrate binding and/or catalysis. However, determination of crystal structures of *Tf*Cel6A with and without a bound substrate analogue did not show conformational changes nor rigidification of this loop upon substrate-binding [29] (Fig 2B, 2D and 2F). In fact, as visible in Fig 2, in the structure of *Tf*Cel6A with a bound substrate analogue, as many as six residues of the N-loop could not be modelled, as opposed to only two or even zero in the ligand-free structures of mgCel6A and *Tf*Cel6A, respectively. Modelling of the substrate-analogue into the catalytic clefts of ligand-free mgCel6A and *Tf*Cel6A (Fig 2) further emphasized the structural variation and the remaining uncertainty concerning the role of Asp79. In the structure of the complex, the closest distance between Asp79 and the scissile bond is 10,6 Å compared to 9,1 Å and 7,7 Å in the models of ligand binding *Tf*Cel6A and mgCel6A, respectively.

### Apparent melting temperature (T_m(app)_)

**Fig 3:**
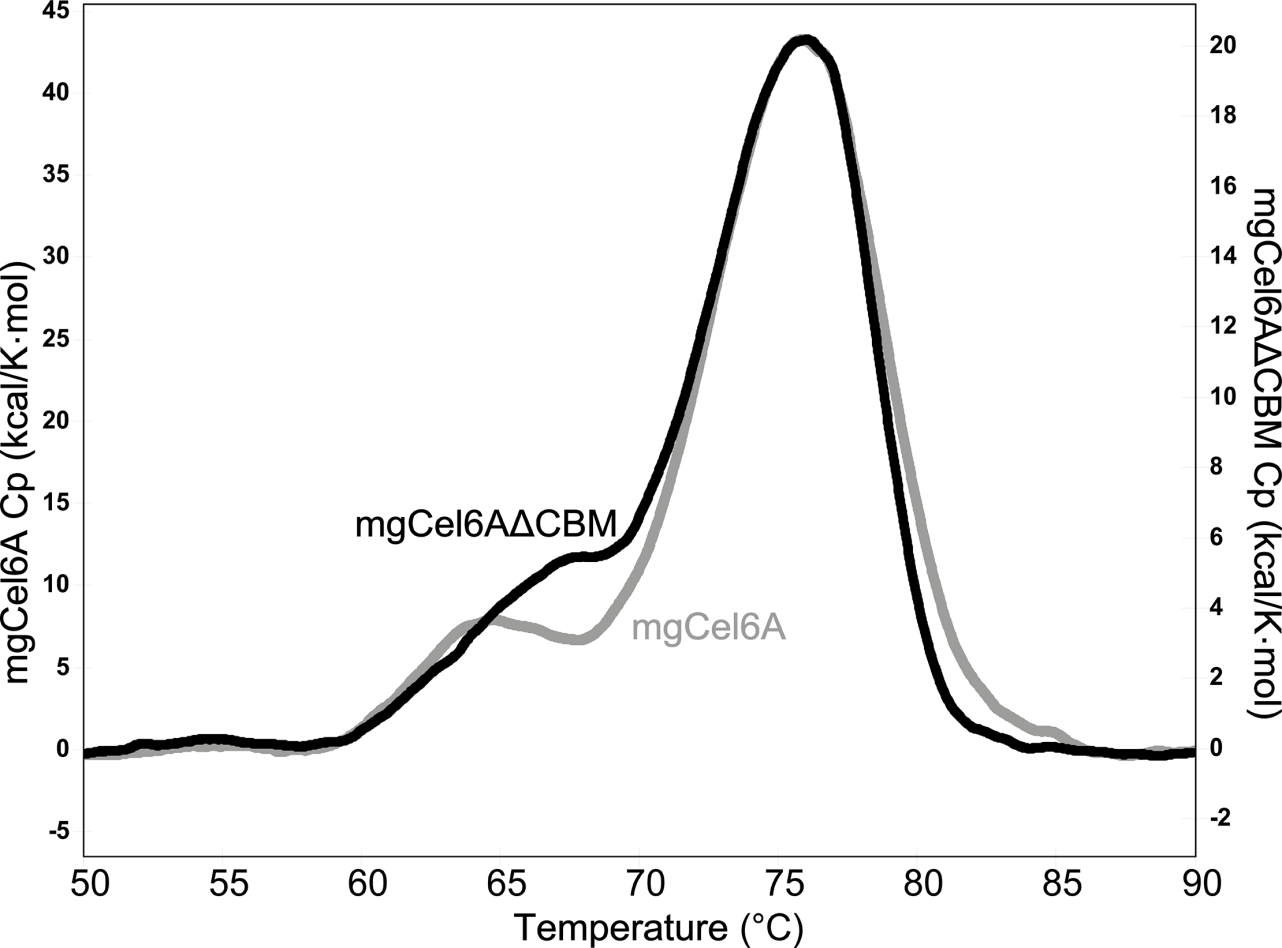
DSC thermograms for mgCel6A and mgCel6AΔCBM. The molar heat capacity (Cp) after buffer baseline subtraction and sigmoidal baseline fitting is plotted against the temperature. The thermograms are displayed as overlay graphs with individual y-axes. The protein samples (pH 6.0) were heated at a rate of 1°C/min.

The apparent melting points of the two enzyme variants mgCel6A and mgCel6AΔCBM were estimated with differential scanning calorimetry (DSC) by monitoring the change in heat capacity along a temperature gradient. Both enzyme variants showed irreversible unfolding, which was not fully two-state, as shown by the shoulders at approximately 65°C (Fig 3). While removal of the CBM had some effect on the shape of this shoulder, the main unfolding event was not affected and both enzyme variants exhibited a T_m(app)_ of 76°C. Since the “shoulder peak” was present for both mgCel6A and mgCel6AΔCBM, it cannot be attributed to independent denaturation of the CBM. It is possible, however, that the shoulder seen for mgCel6A relates to the CBM, whereas the different shoulder seen for mgCel6AΔCBM reflects another irregularity, for example related to fraying at the newly generated C-terminal end. Although DSC data derived from irreversible unfolding processes must not be over-interpreted [50, 51], the data clearly show that mgCel6A and mgCel6AΔCBM are stable enzymes. Notably, preliminary unfolding experiments based on measuring intrinsic fluorescence indicated similar T_m(app)_ values (data now shown).

### Optimal conditions for activity

**Fig 4:**
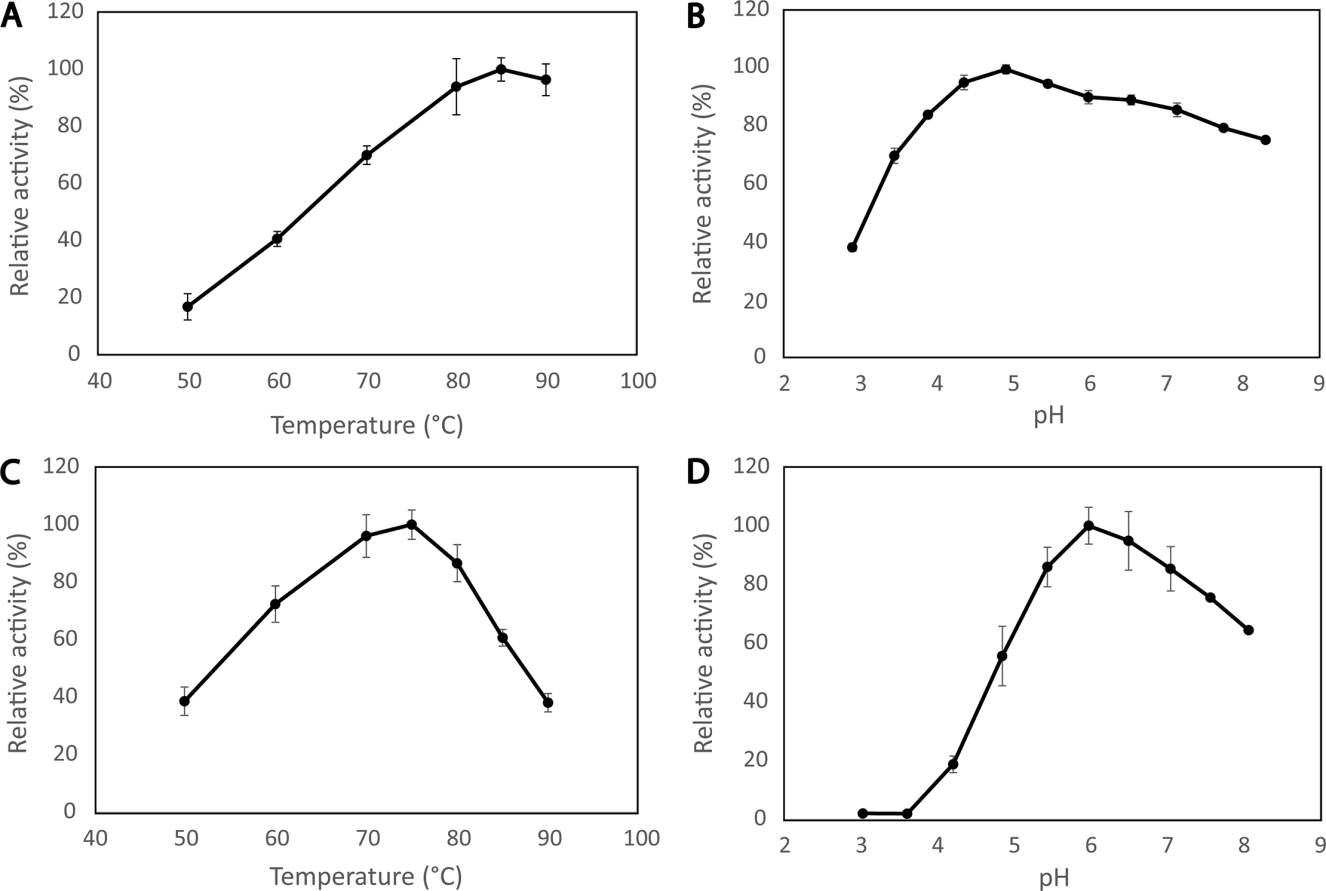
Temperature and pH optimum of mgCel6A. Panels A & B show the optimal temperature at pH 5.0 (A) and the optimal pH at 85°C (B) for activity on CMC, assessed using 10 min reaction times. Panels C & D show the optimal temperature at pH 6.0 (C) and the optimal pH at 75°C (D) for activity on sulfite-pulped spruce, assessed using 15 min reaction times.

When using CMC and short incubation times (10 min), the full-length enzyme performed best at 85°C and pH 5.0 (Fig 4A and 4B). For sulfite-pulped spruce, the optimal reaction conditions were 75°C and pH 6.0 (Fig 4C and 4D). The differences in the optimal temperature and pH displayed in in Fig 4 and the results of similar assays using different conditions (pH, incubation time; not shown) suggest that stability comes into play at temperatures as high as 75°C and pH values below 6.0, as confirmed by the results described below. In any case, the results depicted in Fig 4 confirm that the enzyme is highly thermoactive.

### Operational enzyme stability

**Fig 5:**
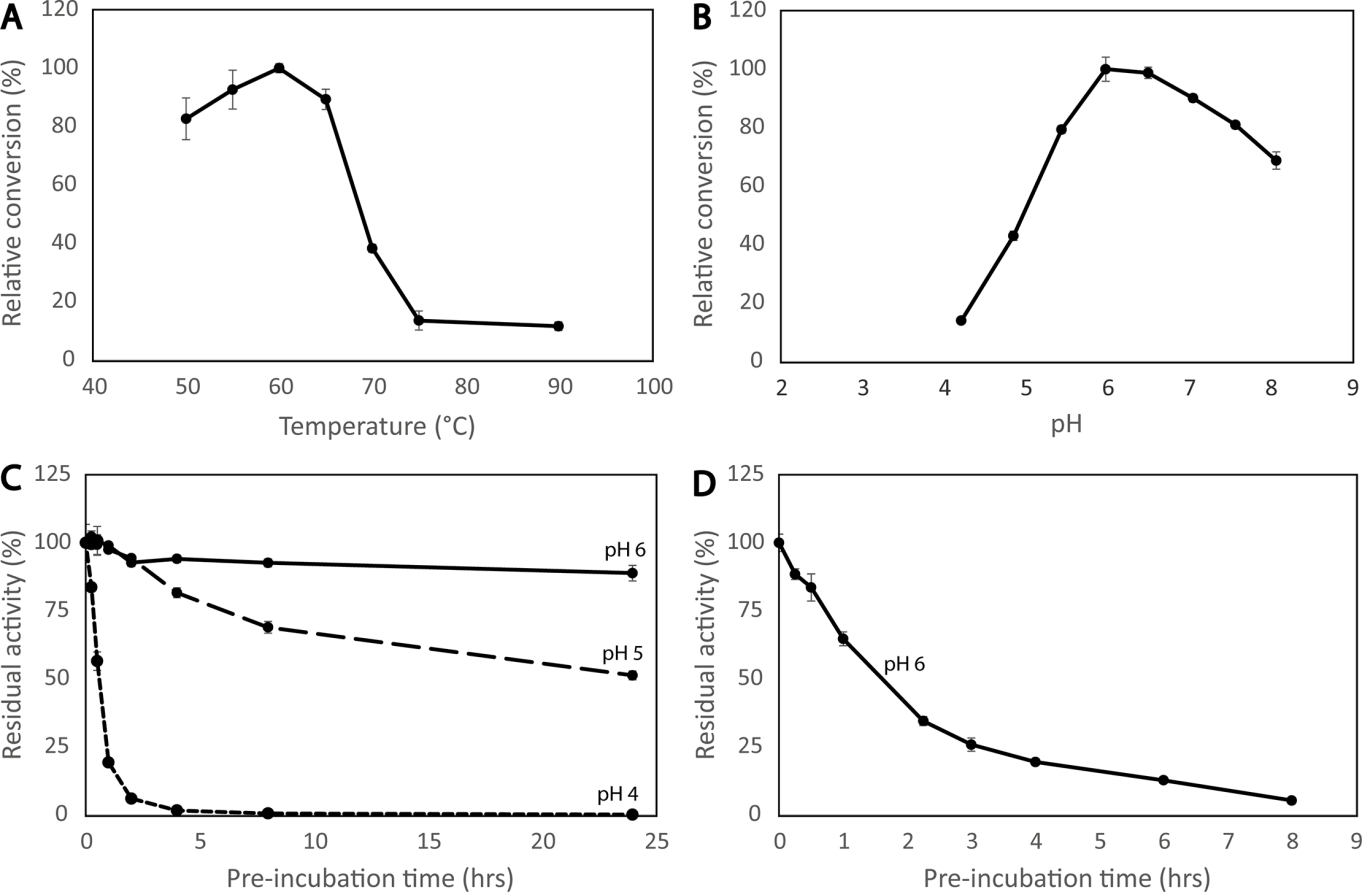
Operational enzyme stability. Panels A & B show the optimal temperature at pH 6.0 (A) and the optimal pH at 60°C (B) for 24 hours reactions with sulfite-pulped spruce. Panel C shows half-life curves which were obtained by pre-incubating the enzyme at pH 4.0, 5.0 or 6.0 at 65°C for 24 hours, followed by measurement of remaining activity in a 10 minute assay with CMC. Panel D shows remaining activity against CMC after incubation of mgCel6A at 70°C, pH 6.0, for various periods of time.

Hydrolysis of sulfite-pulped spruce in overnight reactions (24 hours) showed the highest yields for reactions run at pH 6.0 and 60°C (Fig 5A and 5B). The lower optimum temperature observed here shows that stability comes into play when using more industrially relevant reaction times. Thermal and pH stability were further investigated by constructing half-life curves after pre-incubating the enzyme in various conditions prior to carrying out an activity assay. These experiments showed that the enzyme retained full activity after pre-incubation for 24 hours at 60°C, pH 6.0 and pH 5.0 (results not shown). At higher temperatures or lower pH, the enzyme became unstable. For example, at 65°C the enzyme retained 0%, 50% and 90% activity after 24 hours at pH 4.0, 5.0 or 6.0, respectively (Fig 5C), and at 70°C the half-life of mgCel6A was less than 2 hours when pre-incubated at pH 6.0 (Fig 5D).

### Further studies of enzyme activity

**Fig 6:**
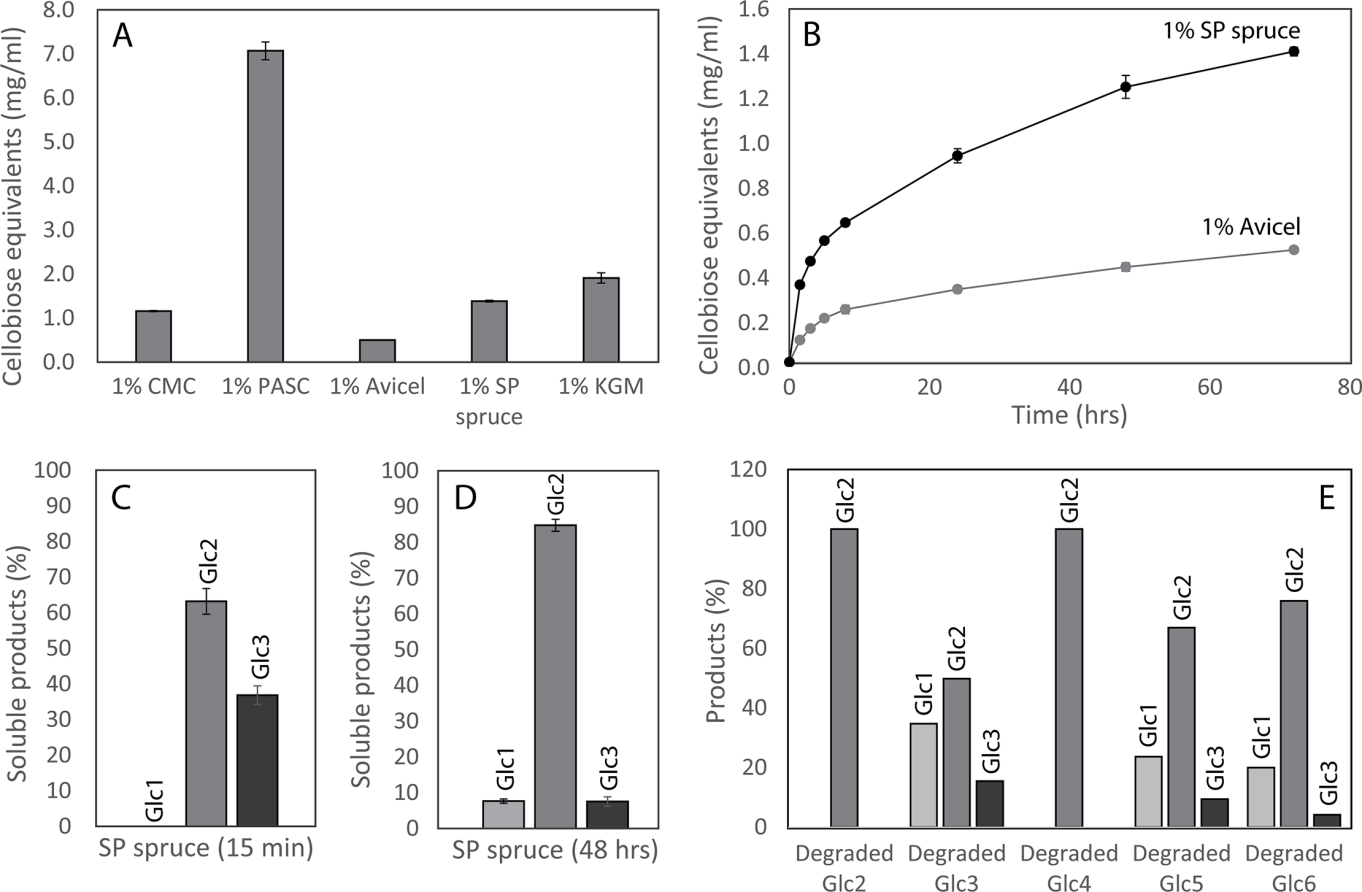
Degradation reactions and hydrolysis products. Panel A shows total solubilization yields after incubating 10 mg/ml CMC, PASC, Avicel, sulfite-pulped spruce and KGM with 1 μM mgCel6A for 72 hours (60°C, pH 6.0). Panel B shows progress curves for hydrolysis of 10 mg/ml sulphite-pulped spruce or Avicel by 1 μM mgCel6A (60°C, pH 6.0). Panel C & D show product profiles from sulfite-pulped spruce (conditions as in A) after 15 minutes (C) and 48 hours (D). Panel E shows product profiles obtained after degrading various soluble cello-oligomers (DP2-DP6, at 0.1 mg/ml), for 18 hours.

The enzyme was active on all the tested cellulosic model substrates (Fig 6A), including CMC, PASC and Avicel which represent various cellulose structures and degrees of crystallinity. CMC is considered an endoglucanase substrate due to the carboxymethyl substitutions not being compatible with the catalytic site (tunnel) of cellobiohydrolases [52]. While indeed active on CMC, mgCel6A did not exhibit high activity on this substrate considering that CMC is a soluble cellulose that should be easily accessible (Fig 6A). Therefore, an additional experiment according to Irwin et al. [40] was carried out, showing that after degradation of filter paper, approximately 40% of the newly generated reducing ends resided in the insoluble substrate. This high fraction of insoluble reducing ends, together with the structural data, clearly show that mgCel6A is an endocellulase. The nature of the endoglucanase mode of action implies that most cuts will be made in the internal parts of the cellulose chains, thereby allowing other enzymes, such as cellobiohydrolases to access the substrate more easily. Although such internal cleavage is crucial for efficient depolymerization of cellulose, endoglucanases alone do not usually lead to a high degree of substrate solubilization, meaning that hydrolysis yields often seem low. MgCel6A alone was able to convert 13.8±0.3% of sulfite-pulped industrial spruce (10% DM) into soluble cello-oligomers during a 72 hours reaction at 60°C, using an enzyme load of 8 mg enzyme per 1 g cellulose.

Progress curves for hydrolysis of sulfite-pulped spruce and Avicel showed that mgCel6A degrades the industrial substrate more easily than Avicel (Fig 6B). While Avicel is predominantly crystalline, the sulfite-pulped spruce likely contains a higher ratio of amorphous regions. Accordingly, compared to Avicel, the enzyme was considerably more efficient on PASC (Fig 6A), a substrate that is primarily amorphous [38]. Thus, mgCel6A seems more active on amorphous cellulose regions, while degrading crystalline cellulose at a slower rate. As commonly observed in enzymatic hydrolysis of cellulose, the reaction rate for mgCel6A became drastically impaired within only a few hours (Fig 6B), suggesting that the substrate rapidly becomes less accessible and degradable after initial fast conversion of the easily accessible parts [53].

### Hydrolysis products

Analysis of reaction products obtained from the industrial substrate showed that mgCel6A generates cellobiose and cellotriose during the initial phase of the reaction (Fig 6C), whereas cellobiose accumulates as the main end-product in longer incubations (Fig 6D). Initial formation of a considerable amount of trimeric products fits well with the notion that mgCel6A is an endo-acting enzyme, whereas the later dominance of the dimer suggests that the trimer is slowly converted. The latter was indeed demonstrated in experiments using cellotriose as substrate (Fig 6E). While the dimer was not cleaved by mgCel6A, all other tested cello-oligomers, from DP3 to DP6, were converted to various amounts of DP1-3, consistent with cellobiose being the dominating end-product (Fig 6E). Notably, Fig 6E also shows that the longer oligomers were converted faster than the trimer.

### Activity on hemicellulose

**Fig 7:**
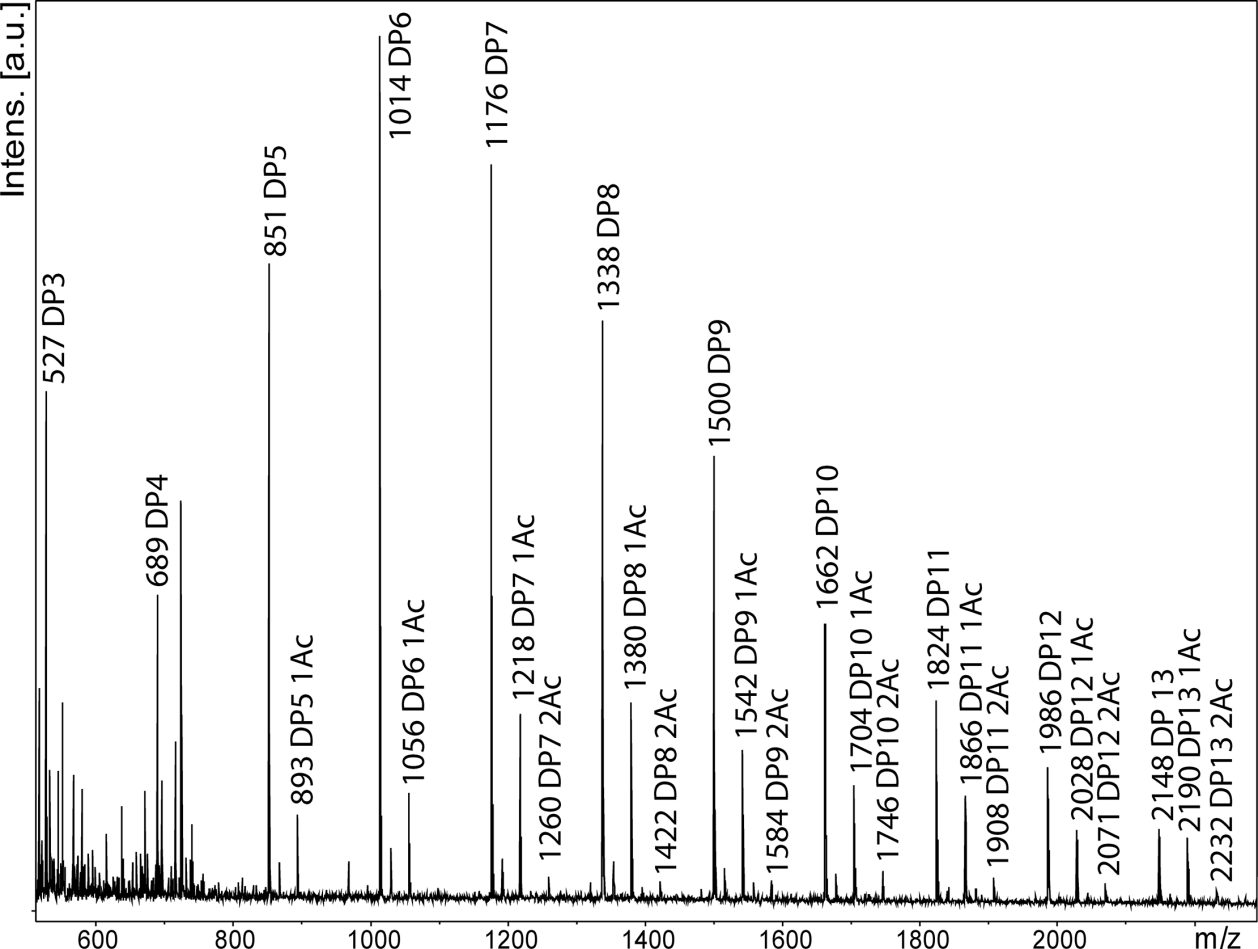
Products generated by mgCel6A from konjac glucomannan (KGM). The indicated m/z values are for sodium adducts of products carrying zero, one (1Ac) or two (2Ac) acetylations. All labelled peaks were not observed in the negative control (= a reaction without enzyme).

The activity of mgCel6A towards various hemicelluloses showed that the enzyme can degrade konjac glucomannan (KGM) (Figs 6A & 7), a hemicellulose consisting of β(1–4) linked glucose and mannan units (with an approximately 40:60 ratio), with backbone acetylations that make the polymer soluble. A mass spectrum of mgCel6A-generated products (Fig 7) showed a variety of products in the DP3-DP13 range, carrying zero, one or two acetylations [54]. KGM is a soluble substrate, which probably offers an explanation to why mgCel6A effectively degrades KGM, relative to the insoluble cellulosic substrates (Fig 6A). Using MS for highly sensitive product analysis, no activity was observed towards xylan or tamarind xyloglucan.

### The effect of the CBM on substrate binding and enzyme efficiency

**Fig 8:**
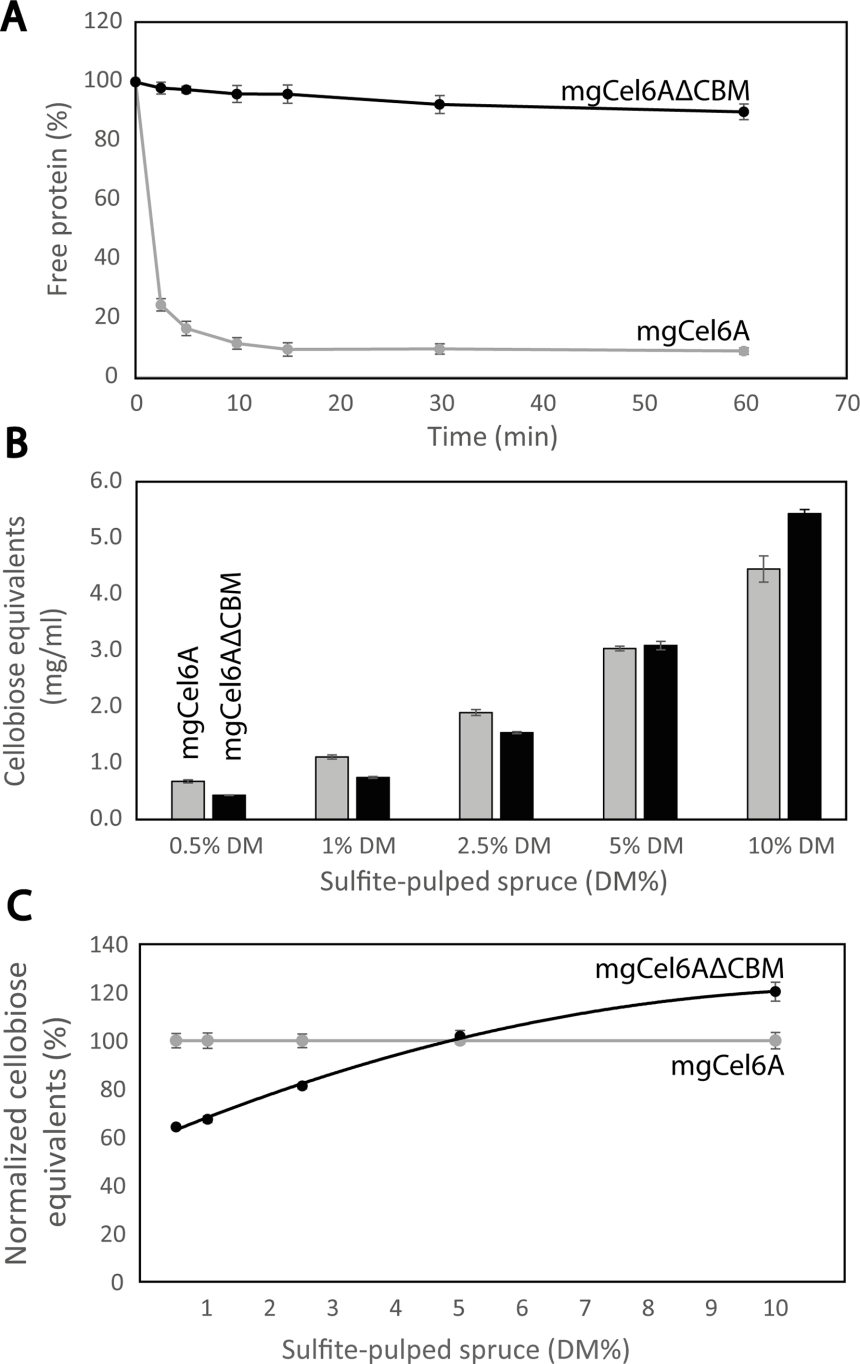
The effect of the CBM on the efficiency of mgCel6A. Panel A shows binding of mgCel6A and mgCel6AΔCBM to Avicel. Panel B shows hydrolysis yields obtained with mgCel6A (grey bars) and mgCel6AΔCBM (black bars), when degrading sulfite-pulped spruce in reactions with various dry matter concentrations (pH 6.0, 60°C, 48 hrs). The enzyme concentration was kept constant at 1 μM, independently of substrate concentration. Panel C shows a comparison of normalized hydrolysis yields obtained with mgCel6AΔCBM or mgCel6A at various substrate concentrations. At each dry matter concentration, the yield obtained with the full-length enzyme was set to 100%.

Substrate binding assays with Avicel showed that removal of the CBM from mgCel6A drastically reduced substrate affinity (Fig 8A). Whereas most of the full-length enzyme was bound to the substrate after a few minutes of incubation, most of the truncated variant remained in solution even after longer incubations. This is an expected result, that has, for example, also been described for fungal cellulases [55].

The closest characterized homolog of mgCel6A, *Tf*Cel6A, also has a CBM2 (although with only 34% sequence identity to the CBM2 discussed in this paper), which has been shown to increase binding affinity to insoluble substrates and to increase hydrolysis yields at low substrate concentrations (~1% DM) [40]. Likewise, at low substrate concentrations (0.5–2.5% DM), hydrolysis yields obtained with mgCel6AΔCBM were only 60–80% of the yields obtained with the full-length enzyme. However, at higher substrate concentrations, there was no difference and at 10% DM, mgCel6AΔCBM outperformed mgCel6A (Fig 8B and 8C). Thus, at industrially relevant substrate concentrations, it seems advantageous to employ this GH6 without its CBM2 in order to maximize the yield.

Interestingly, a previous study on fungal CBM1-containing fungal cellulases has shown that, while the CBM1-containing enzymes are clearly more efficient at low substrate concentrations, they are outperformed by their truncated, CBM1-free variants at high substrate concentrations (which imply low water contents). The present data, for a bacterial CBM2, indicate that the substrate concentration-dependence of the impact of CBMs is a general phenomenon. One explanation is that at high substrate concentrations the “proximity effects” ascribed to CBMs are superseded by the substrate concentration being saturating. In addition, CBMs likely hamper the rate of substrate desorption, which, under conditions where the CBM no longer contributes to the rate of substrate binding will lead to a net reduction in the overall reaction rate [55, 56].

### Concluding remarks

By mining metagenomic data originating in a relevant natural biodiversity, we have obtained and characterized a thermostable bacterial GH6 cellulase, for which structural and functional data demonstrate endoglucanase activity. The enzyme is active on all tested cellulosic substrates, including industrial sulfite-pulped spruce. The overall solubilization yield for the latter was approximately 14% when loaded at 8 mg/g cellulose, which is a promising yield for an endoglucanase acting alone on insoluble cellulose [57, 58].

MgCel6A resembles the well-known *Tf*Cel6A endoglucanase in several ways, but has a seemingly higher temperature optimum than the 55–58°C that has been reported for the *T*. *fusca* enzyme [33, 59] (note that assay conditions affect the apparent temperature optimum, complicating direct comparison with literature data). Importantly, mgCel6A seems much easier to produce in E. coli (500 mg/L for the full-length enzyme, versus 30–40 mg/L reported for *Tf*Cel6A; [60]). The latter is quite remarkable considering that mgCel6A contains a 40-residue P/T linker. *Tf*Cel6A is known to be active on CMC, PASC and filter paper; it is not known whether the enzyme, like mgCel6A, is also active on glucomannan. Enzyme assays with *Tf*Cel6A are commonly performed at 50–55°C [40, 46, 47, 61]. MgCel6A shows good operational stability at 60°C in overnight reactions with sulfite-pulped spruce, a T_m(app)_ of 76°C according to DSC, and the enzyme retains 90% activity after pre-incubation at 65°C for 24 hours (Calza et al showed that *Tf*Cel6A loses less than 20% of its activity after 18 hours at 56°C [59]).

In conclusion, it seems that mgCel6A is a useful enzyme for conducting studies of cellulose degradation at elevated temperatures. This novel endoglucanase exhibits desirable properties compatible with industrial process conditions and has activity on an industrial lignocellulosic substrate, making it a promising enzyme for development of industrial cellulose conversion processes. When employing mgCel6A at industrially relevant substrate concentrations (≥10% DM), the hydrolysis yield was enhanced by removal of the CBM. The true potential of mgCel6A will be explored in follow-up studies, where the enzyme will be assessed in the context of synergistic enzyme cocktails.
